# Survival Results by the Prospectively Determined Clinical Staging for Locally Advanced Gastric Cancer: A Follow‐Up Study of JCOG1302A

**DOI:** 10.1002/ags3.70293

**Published:** 2026-10-06

**Authors:** Tsutomu Hayashi, Ryosuke Kato, Noriko Mitome, Rei Ogawa, Takeo Fukagawa, Hitoshi Katai, Souya Nunobe, Masanori Tokunaga, Etsuro Bando, Yuichi Ito, Takanobu Yamada, Takashi Nomura, Shigeto Makino, Takahiro Kinoshita, Hisashi Hara, Masaki Aizawa, Narikazu Boku, Yukinori Kurokawa, Masanori Terashima, Takaki Yoshikawa

**Affiliations:** ^1^ Department of Gastric Surgery National Cancer Center Hospital Tokyo Japan; ^2^ JCOG Data Center/Operations Office National Cancer Center Hospital Tokyo Japan; ^3^ Department of Surgery Teikyo University School of Medicine Tokyo Japan; ^4^ Department of Gastrointestinal Surgery Tachikawa Hospital Tokyo Japan; ^5^ Gastric Surgery Department Cancer Institute Hospital of JFCR Tokyo Japan; ^6^ Division of Gastric Surgery Shizuoka Cancer Center Shizuoka Japan; ^7^ Department of Gastroenterological Surgery Aichi Cancer Center Hospital Nagoya Japan; ^8^ Department of Gastrointestinal Surgery Kanagawa Cancer Center Yokohama Japan; ^9^ Department of Surgery Yamagata Prefectural Central Hospital Yamagata Japan; ^10^ Department of Surgery Nagaoka Chuo General Hospital Niigata Japan; ^11^ Department of Gastric Surgery National Cancer Center Hospital East Kashiwa Japan; ^12^ Department of Gastroenterological Surgery Sakai City Medical Center Sakai Japan; ^13^ Department of Gastrointestinal Surgery Niigata Cancer Center Hospital Niigata Japan; ^14^ Department of Medical Oncology Institute of Medical Science, University of Tokyo Tokyo Japan; ^15^ Department of Gastroenterological Surgery Osaka University Graduate School of Medicine Osaka Japan; ^16^ Department of Gastrointestinal Surgery Yokohama City University Yokohama Japan

**Keywords:** clinical staging, gastric cancer, multicenter study, preoperative diagnosis, survival analysis

## Abstract

**Background:**

Long‐term overall survival (OS) of gastric cancer stratified by the prospectively determined clinical stage remains unclear.

**Methods:**

This is an ancillary study of JCOG1302A, a prospective observational study that evaluated the accuracy of clinical diagnosis of gastric cancer with cT2/T3/T4, cN0/N1/N2/N3, and M0. The cT diagnosis was comprehensively determined by upper gastrointestinal endoscopy and upper abdominal contrast CT scan with 1 or 5 mm slice thickness. Lymph nodes with a minor axis greater than 8 mm or a major axis greater than 10 mm were defined as positive for metastasis. This follow‐up study evaluated OS in eligible patients from JCOG1302A.

**Results:**

Among 1 260 patients enrolled in JCOG1302A between July 2013 and November 2014, survival data of 1 176 were analyzed. With a median follow‐up of 6.0 years for 821 surviving patients, the 5 year‐OS was 82.1% (95% CI, 77.3–85.9) in cT2 (*n* = 319), 72.7% (68.2–76.6) in cT3 (*n* = 450), 60.0% (54.9–64.7) in cT4a (*n* = 401), and 40.0% (5.2–75.3) in cT4b (*n* = 6), while that was 78.0% (74.2–81.2) in cN0 (*n* = 559), 70.6% (65.4–75.2) in cN1 (*n* = 350), 59.1% (52.5–65.1) in cN2 (*n* = 241), and 28.4% (12.7–46.5) in cN3 (*n* = 26). When combined with cT and cN, 5y‐OS was 83.5% (77.8–87.8) in cT2N0 (*n* = 226), 77.2% (71.0–82.2) in cT3N0 (*n* = 232), 66.8% (56.3–75.2) in cT4aN0 (*n* = 100), 78.7% (68.6–85.8) in cT2N(+) (*n* = 93), 68.0% (61.1–73.8) in cT3N(+) (*n* = 218), and 57.7% (51.7–63.2) in cT4aN(+) (*n* = 301).

**Conclusion:**

Clinical T and N categories were associated with OS in locally advanced gastric cancer, providing prognostic information that may help identify higher‐risk clinical subgroups.

## Graphical abstract

Prospectively determined clinical T and N categories clearly stratified long‐term survival in locally advanced gastric cancer. Patients with cT3N+, cT4aN0, cT4aN+, and cT4b disease showed unfavorable prognosis despite standard treatment. These findings may help optimize patient selection for perioperative treatment strategies.

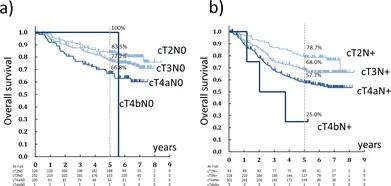

## 1 Introduction

For resectable advanced gastric cancer, multi‐modality treatment including surgery and chemotherapy is a standard approach. Several Phase III studies clearly showed survival benefits with pre‐ and post‐operative chemotherapy [1, 2, 3]. However, it remains controversial which patients are optimal candidates for perioperative chemotherapy among all resectable locally advanced gastric cancer based on the clinical diagnosis, particularly in Japan, for two reasons [1]: a high proportion of early disease which looks like locally advanced disease due to inflammatory changes makes it difficult to correctly select advanced disease, resulting in limited accuracy of clinical diagnosis, and [2] relatively long overall survival is achieved only by adjuvant chemotherapy whose indication is determined precisely on the basis of pathological findings after upfront surgery [4]. However, there is much room to improve prognosis of pathological Stage III even in Japan. Considering low compliance with intensive chemotherapy after surgery, preoperative chemotherapy is attractive to further improve survival.

To select patients eligible for neoadjuvant chemotherapy (NAC), we evaluated the diagnostic accuracy of the clinical T and N categories by endoscopy and computed tomography (CT) in a prospective multicenter observational study (JCOG1302A) [5]. As previously reported, contamination of pathological Stage I was found in 50.4% in cT2N0, 38.7% in cT2N(+), 26.7% in cT3N0, 10.6% in cT3N(+), 9.0% in cT4aN0, and 3.0% in cT4aN(+), while proportion of pathological Stage III was included in 15% in cT3N0, 34% in cT3N+, 8% in cT4aN0, and 38% in cT4aN+. Based on these results, we concluded that cT3N(+) and cT4N(+) were the most optimal candidates for preoperative chemotherapy, and thereafter initiated a Phase III trial (JCOG1509) [6].

Although clinical Stage III would be the optimal target for NAC, this approach must be validated based on actual survival outcomes to provide information that may help select the optimal candidate for perioperative treatment strategies based on clinical stage. While treatment decisions of adjuvant chemotherapy for resectable advanced gastric cancer have traditionally been based on pathological staging, accurate clinical staging to minimize the risk of overtreatment has gained increasing attention in the era of increasing adoption of NAC. However, no study has examined long‐term survival stratified by prospectively determined clinical stage. Therefore, the primary objective of this follow‐up study was to describe long‐term overall survival according to prospectively determined clinical T and N categories and their combinations in patients enrolled in JCOG1302A. The prognostic associations of clinical T and N categories with overall survival were also evaluated.

## 2 Patients and Methods

### 2.1 Study Design

This is an ancillary study of JCOG1302A, which was a prospective multicenter observational study conducted by the Stomach Cancer Study Group of the Japan Clinical Oncology Group (JCOG). The eligibility criteria of JCOG1302A were as follows: (1) histologically proven gastric adenocarcinoma; (2) cT2 (proper muscle layer) or deeper invasion diagnosed either by gastric endoscopy or by contrast‐enhanced CT; (3) neither linitis plastica (Borrmann Type 4) nor diffuse ulcerative tumor (Type 3) with diameter of 8 cm or larger; (4) esophageal invasion < 3 cm; (5) no paraaortic or extensive bulky lymph node metastases (solitary node larger than 3 cm or at least two adjacent nodes larger than 1.5 cm along the celiac, splenic, common, or proper hepatic arteries); (6) no evidence of Stage IV disease; (7) no prior staging laparoscopy; (8) no previous gastrectomy, chemotherapy, or radiotherapy for gastric cancer. Preoperative clinical diagnoses for the tumor invasion (cT category) and lymph node metastasis (cN category) were prospectively staged according to the 14th edition of the Japanese Classification of Gastric Carcinoma. In this study (JCOG1302A2), follow‐up data were collected additionally.

### 2.2 Criteria for Clinical Diagnosis of cT/cN Categories in JCOG1302A

Clinical T and N were principally determined by multidetector row CT (MDCT) using more than four detector rows with a slice thickness of 1 or 5 mm. cT1 disease was excluded by gastric endoscopy, and T category was diagnosed on CT as follows: T2: Localized wall thickening with contrast enhancement but smooth serosal surface, T3: Diffuse wall thickening with contrast enhancement but smooth serosal surface, T4a: Diffuse wall thickening with contrast enhancement, with irregular or nodular change of serosal surface, and/or infiltration of perigastric fatty tissue, and T4b: Loss of fat layer between surrounding organs and the gastric tumor. Typical CT images for each T stage were shown in the protocol to support the diagnosis.

All the lymph nodes having either a minor axis ≥ 8 mm or a major axis ≥ 10 mm were diagnosed as metastasis, and their nodal size and anatomic location (station numbers) were recorded. The lymph nodes not satisfying the aforementioned criteria but clinically suspicious of metastasis were also recorded with their size and location. When the lymph nodes were aggregated and diagnosed as metastasis, the size and location of the individual nodes were recorded.

For the analysis combining clinical T and N categories, cN1–3 disease was grouped as cN(+) to explore a clinically applicable risk stratification based on the combination of tumor depth and the presence or absence of clinically detected nodal metastasis.

### 2.3 Surgery and Postoperative Chemotherapy

Within 35 days of enrollment, patients underwent laparotomy. When the disease was judged resectable, gastrectomy with D2 lymph node dissection was performed following the third version of the Japanese Gastric Cancer Treatment Guidelines of the Japanese Gastric Cancer Association (JGCA) [7]. Pathological examination of the resected specimens was performed in each institution on the basis of the 14th edition of the Japanese Classification of Gastric Carcinoma [8]. The administration of postoperative chemotherapy was determined by each physician based on JGCA guidelines [7].

### 2.4 Follow‐Up

Patients were followed up at each registered hospital as part of clinical practice. The survival data and administration of adjuvant chemotherapy were collected at the JCOG Data Center.

### 2.5 Statistical Considerations

Overall survival was defined as the time from enrollment to death from any cause. Survival curves were estimated by the Kaplan–Meier method. Confidence intervals for the proportion of patients alive at T years were estimated using Greenwood's formula. Hazard ratios (HRs) and 95% confidence intervals (CIs) for overall survival were estimated using Cox proportional hazards models. A multivariable Cox proportional hazards model included clinical T category, clinical N category, age, sex, tumor location, and pretreatment histological type. All statistical analyses were performed using SAS, version 9.4.

### 2.6 Ethical Issue

This study protocol was approved by the JCOG Protocol Review Committee and the institutional review board of each participating hospital before initiation of the study. This study was conducted in accordance with the international ethical recommendations stated in the Declaration of Helsinki and Ethical Guidelines for Medical and Health Research Involving Human Subjects. The JCOG Data Center conducted central monitoring to ensure data submission, patient eligibility, and on‐schedule study progress.

## 3 Results

### 3.1 Baseline Characteristics

Between July 2013 and November 2014, 1 275 patients were enrolled in JCOG1302A from 53 Japanese specialized institutions. Of these, eight patients did not undergo surgery for reasons including unfitness for surgery and withdrawal of consent, and seven did not fulfill the eligibility criteria (Type 4 or large Type 3 in 3 patients, malignant lymphoma diagnosed by intraoperative frozen section of lymph node in 2, and multiple advanced gastric tumors in 2). Thus, 1 260 patients were eligible for the primary analysis of this study to evaluate the accuracy of clinical diagnosis in JCOG1302A.

Moreover, among the 1 260 patients, 84 patients were excluded from this ancillary study due to loss to follow‐up in 83 patients and cT1b in 1. Finally, survival was analyzed in 1 176 patients. The patient flow diagram is shown in Figure 1. The baseline characteristics of these patients are summarized in Table 1. Limited lymph node dissection was performed in 5 patients; proximal gastrectomy in 4 patients, and pylorus‐preserving gastrectomy in 1. Exploratory laparotomy without resection was performed in 27 patients due to peritoneal metastasis found during surgery. Finally, 1 149 patients (98%) underwent gastrectomy with D2 lymph node dissection.

**FIGURE 1:**
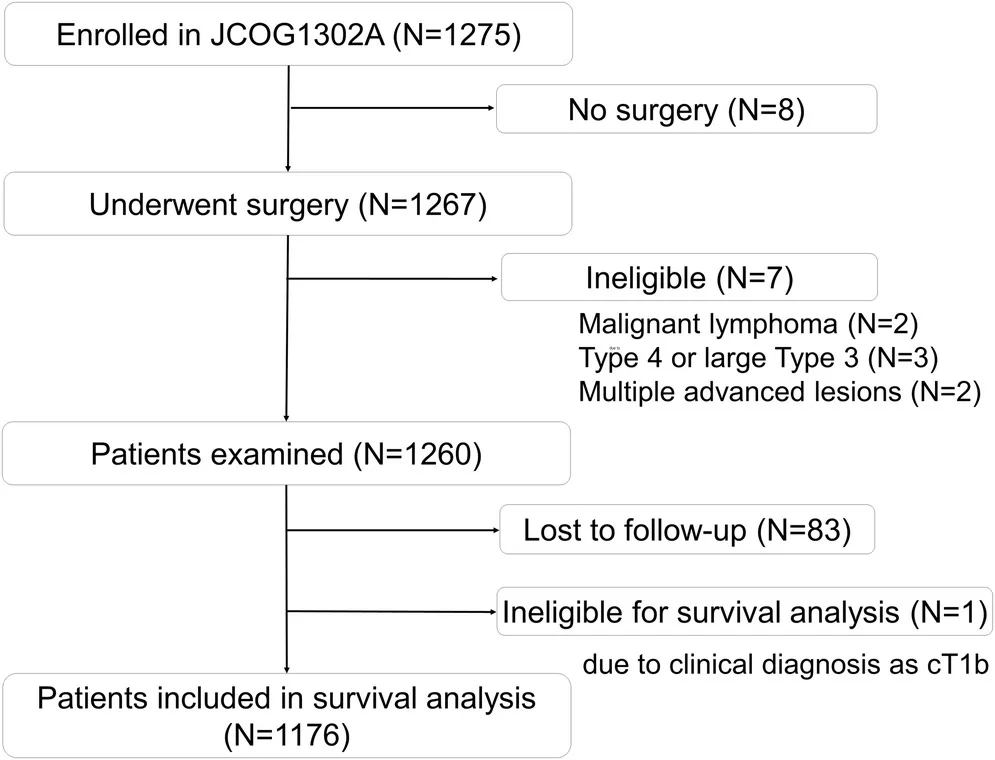
Patient flow diagram of the study cohort. A total of 1 275 patients were enrolled in the JCOG1302A trial. Among them, 1 267 patients underwent surgery and 1 260 were examined. After excluding patients who did not undergo surgery (*n* = 8), those who were ineligible (*n* = 7; malignant lymphoma, *n* = 2; Type 4 or large Type 3 tumors, *n* = 3; multiple advanced lesions, *n* = 2), those lost to follow‐up (*n* = 83), and one patient ineligible for survival analysis due to a clinical diagnosis of cT1b, 1 176 patients were included in the survival analysis.

**TABLE 1:** Clinicopathological characteristics (*n* = 1176).

Characteristics	Number of patients (%)
Age, median (range)	68 years (29–92)
Sex	
Male	793 (67)
Female	383 (33)
ECOG‐PS	
0	923 (78)
1	207 (18)
2	40 (3)
3	6 (0.5)
Charlson morbidity index	
0	879 (75)
1	223 (19)
2	56 (5)
3	14 (1)
4	4 (0.3)
Tumor location	
Upper third	250 (21)
Middle third	427 (36)
Lower third	499 (42)
Histological type	
Differentiated	679 (58)
Undifferentiated	495 (42)
Unknown	2 (0.1)
Surgical procedure	
Total gastrectomy	336 (29)
Distal gastrectomy	808 (69)
Proximal gastrectomy	4 (0.3)
Pylorus‐preserving gastrectomy	1 (0.1)
Laparotomy only	27 (2)
Adjuvant chemotherapy	
Presence	615 (52)
Absence	561 (48)

Table 2 shows the number of patients stratified by the combination of cT and cN according to the 14th edition of the Japanese Classification of Gastric Carcinoma. The proportion of each TN category was almost concordant with our previous reports [5].

**TABLE 2:** Distribution in cT and cN categories according to TNM 7th and 8th classification.

Characteristics	cN0 (0)	cN1* (1–2)	cN2* (3–6)	cN3* (7‐)	cN + **	Total
cT2 (MP)	226	75	18	0	93	319
cT3 (SS)	232	120	89	9	218	450
cT4a (SE)	100	153	132	16	301	401
cT4b (SI)	1	2	2	1	5	6
Total	559	350	241	26	617	1 176

* TNM 7th edition.

** TNM 8th edition.

Table 3 demonstrates the details of the adjuvant chemotherapy stratified by final pathological stage. Briefly, adjuvant chemotherapy was initiated in approximately 70% of patients at Stage II or more. S‐1 monotherapy was selected in approximately 80% of patients who received adjuvant chemotherapy. The administration of adjuvant chemotherapy according to the combined clinical T and N categories is shown in the Table S1. The proportion of patients receiving adjuvant chemotherapy differed among clinical subgroups, ranging from 34.5% in cT2N0 to 65.1% in cT4aN(+), excluding the small cT4b subgroups.

**TABLE 3:** Details of adjuvant chemotherapy.

Stage* (Number of patients)	Number of patients (%)				
	Total**	S‐1	DS***	Other	Unknown
Stage IA (112)	0 (0)	0	0	0	0
Stage IB (141)	8 (6)	7	0	1	0
Stage IIA (196)	73 (37)	71	0	2	0
Stage IIB (173)	117 (68)	117	0	0	0
Stage IIIA (152)	114 (75)	99	6	9	0
Stage IIIB (153)	123 (80)	107	5	10	1
Stage IIIC (125)	95 (76)	78	6	11	0
Stage IV (123)	84 (68)	44	4	36	0
Unknown (1)	1 (100)	1	0	0	0
Total (1 176)	615 (52)	524	21	69	1

* The 14th edition of the Japanese Classification of Gastric Carcinoma.

** Numbers in parentheses were the proportion who received adjuvant chemotherapy.

*** Docetaxel plus S‐1.

### 3.2 Survival Outcome According to the cT and cN

Survival curves stratified by cT and cN following TNM 7th edition are shown in Figs. 2 and 3, respectively. With a median follow‐up of 6.0 years (IQR 5.0–6.9) for 821 surviving patients, the overall survival at 5 years was 82.1% (95% CI, 77.3%– 85.9%) in cT2 (*n* = 319), 72.7% (68.2%–76.6%) in cT3 (*n* = 450), 60.0% (54.9%– 64.7%) in cT4a (*n* = 401), and 40.0% (5.2%–75.3%) in cT4b (*n* = 6), while that was 78.0% (74.2%–81.2%) in cN0 (*n* = 559), 70.6% (65.4–75.2) in cN1 (*n* = 350), 59.1% (52.5–65.1) in cN2 (*n* = 241), and 28.4% (12.7–46.5) in cN3 (*n* = 26). The survival curves were clearly separated by cT. Although the survival curves of cN2 and cN3 were well separated, those of cN0 and cN1 were less clearly separated. Using cT2 as the reference, the hazard ratios (HRs) for overall survival were 1.560 (95% CI, 1.151–2.113) for cT3, 2.534 (1.891–3.396) for cT4a, and 5.588 (2.033–15.357) for cT4b. Using cN0 as the reference, the corresponding HRs were 1.297 (1.004–1.676) for cN1, 2.038 (1.573–2.641) for cN2, and 4.898 (2.990–8.022) for cN3 (Table 4).

**FIGURE 2:**
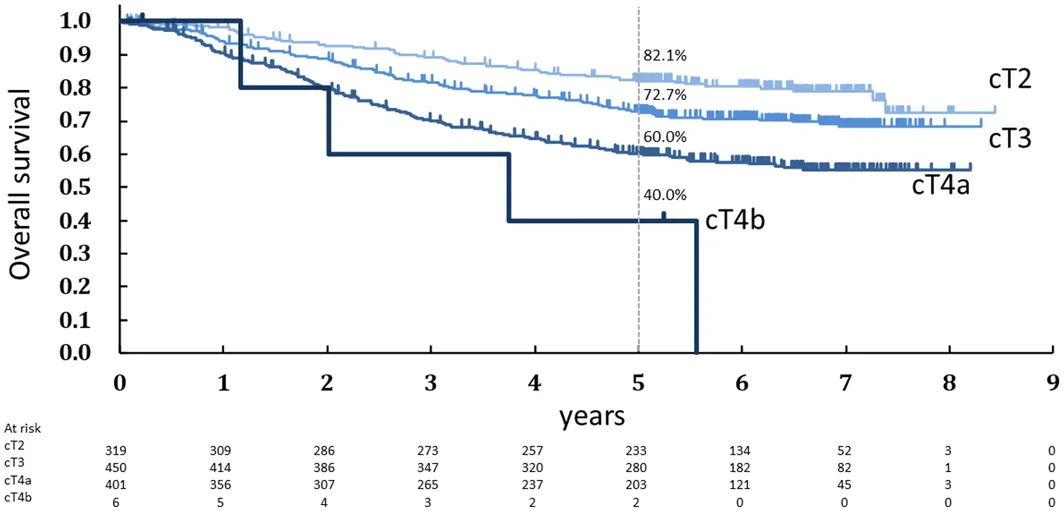
Overall survival according to clinical T category. Kaplan–Meier curves showing overall survival stratified by clinical T stage (cT2, cT3, cT4a, and cT4b). The number of patients at risk at each time point is shown below the graph.

**FIGURE 3:**
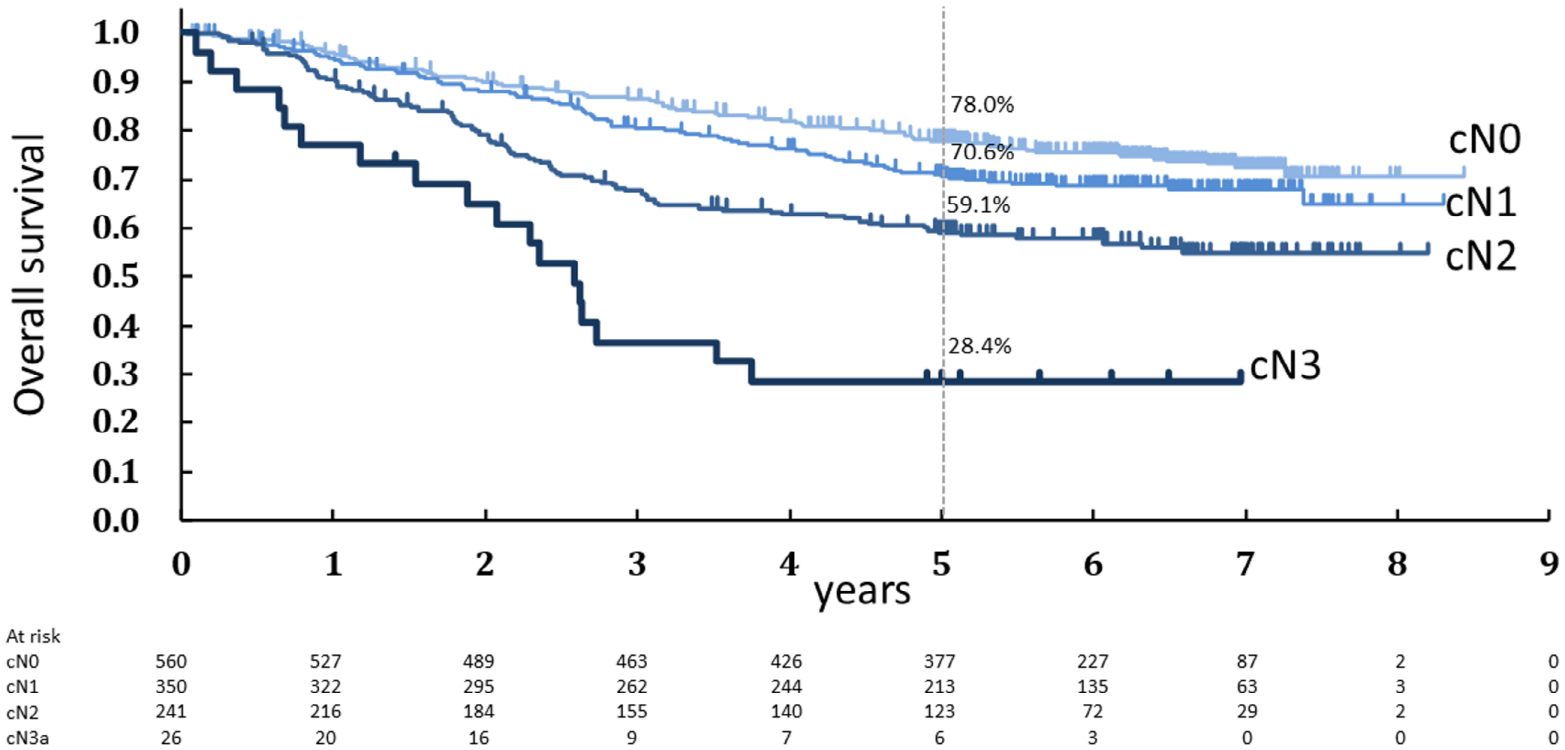
Overall survival according to clinical N category. Kaplan–Meier curves showing overall survival stratified by clinical nodal status (cN0, cN1, cN2, and cN3). The number of patients at risk at each time point is shown below the graph.

**TABLE 4:** Univariable Cox proportional hazards analyses for overall survival according to clinical T category, clinical N category, and their combinations.

Clinical category	Hazard ratio	95% CI	*p*
Clinical T category			
cT2	1.000 (reference)	—	—
cT3	1.560	1.151–2.113	0.0041
cT4a	2.534	1.891–3.396	< 0.0001
cT4b	5.588	2.033–15.357	0.0009
Clinical N category			
cN0	1.000 (reference)	—	—
cN1	1.297	1.004–1.676	0.0467
cN2	2.038	1.573–2.641	< 0.0001
cN3	4.898	2.990–8.022	< 0.0001
Combined clinical T/N category			
cT2N0	1.000 (reference)	—	—
cT2N(+)	1.254	0.741–2.122	0.3987
cT3N0	1.408	0.943–2.104	0.0946
cT3N(+)	1.981	1.347–2.913	0.0005
cT4aN0	2.220	1.414–3.486	0.0005
cT4aN(+)	2.898	2.039–4.121	< 0.0001

Abbreviation: CI, confidence interval.

### 3.3 Survival Outcome According to the Combination of cT and cN

Survival curves stratified by the combination of cT and cN are shown in Figure 4a (cN0 cases) and Figure 4b (cN+ cases). The overall survival at 5 years was 83.5% (77.8–87.8) in cT2N0 (*n* = 226), 78.7% (68.6–85.8) in cT2N+ (*n* = 93), 77.2% (71.0–82.2) in cT3N0 (*n* = 232), 68.0% (61.1–73.8) in cT3N+ (*n* = 218), 66.8% (56.3–75.2) in cT4aN0 (*n* = 100), and 57.7% (51.7–63.2) in cT4aN+ (*n* = 301). The overall survival at 5 years of cT4aN0 was remarkably poorer than that of cT3N0. Using cT2N0 as the reference, the HRs for overall survival were 1.254 (95% CI, 0.741–2.122) for cT2N(+), 1.408 (0.943–2.104) for cT3N0, 1.981 (1.347–2.913) for cT3N(+), 2.220 (1.414–3.486) for cT4aN0, and 2.898 (2.039–4.121) for cT4aN(+). Thus, cT3N(+) and cT4aN0 showed a similar magnitude of increased mortality risk relative to cT2N0, whereas cT3N0 did not show a statistically significant increase in risk (Table 4).

**FIGURE 4:**
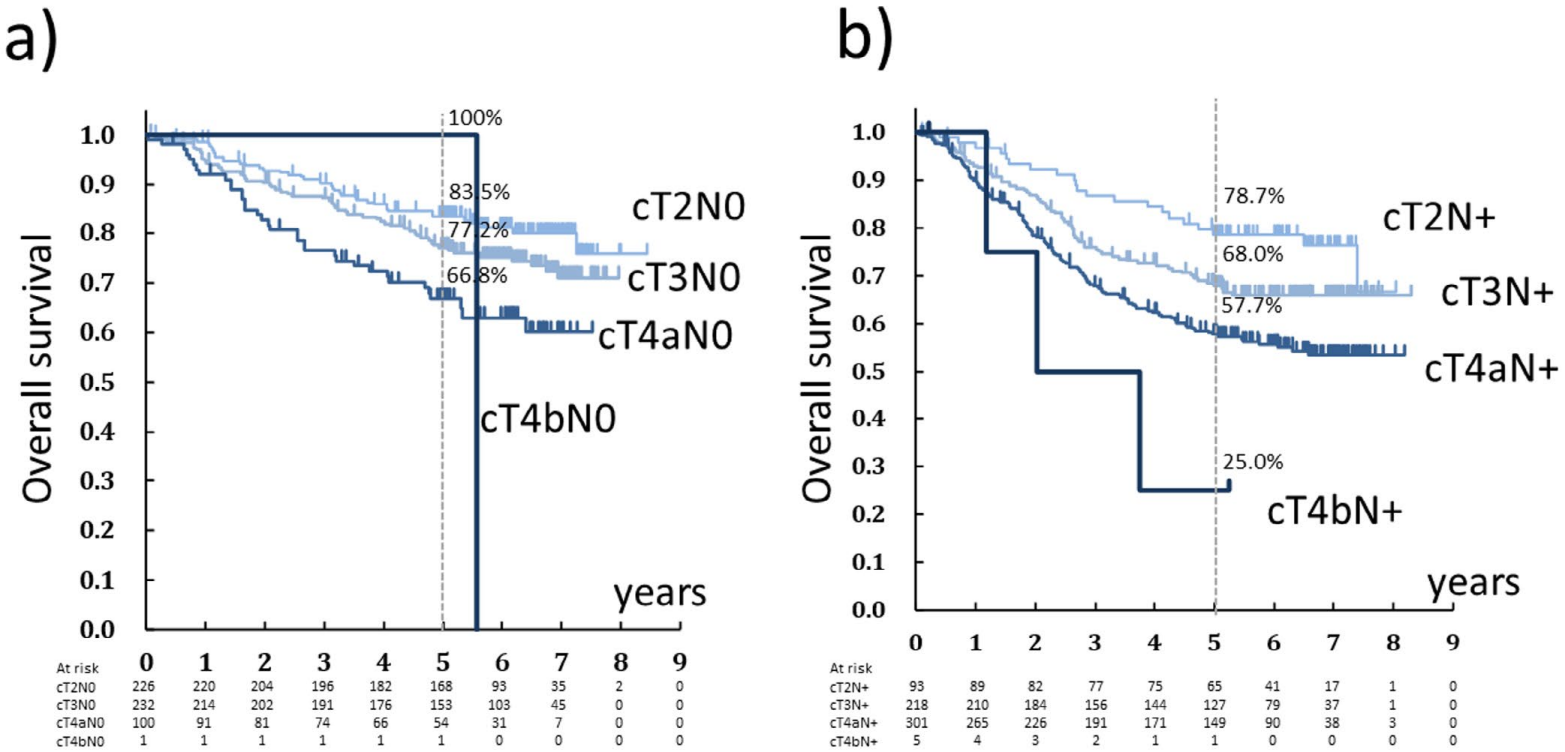
Overall survival according to combined clinical T and N categories. Kaplan–Meier curves showing overall survival stratified by combined clinical T and N categories. (a) Survival curves for node‐negative patients (cN0) according to T stage (cT2N0, cT3N0, cT4aN0, and cT4bN0). (b) Survival curves for node‐positive patients (cN+) according to T stage (cT2N+, cT3N+, cT4aN+, and cT4bN+). The number of patients at risk at each time point is shown below the graphs.

### 3.4 Multivariable Analysis for Overall Survival

In the multivariable Cox proportional hazards analysis including clinical T category, clinical N category, age, sex, tumor location, and pretreatment histological type, cT4a disease (HR, 1.894; 95% CI, 1.380–2.599; *p* < 0.0001), cN2 disease (HR, 1.628; 95% CI, 1.231–2.152; *p* = 0.0006), and cN3 disease (HR, 3.338; 95% CI, 1.986–5.612; *p* < 0.0001) were significantly associated with poorer overall survival. Older age was also associated with poorer overall survival, whereas differentiated histological type was associated with better overall survival (Table 5).

**TABLE 5:** Multivariable Cox proportional hazards analysis for overall survival.

Variable	Category	Adjusted hazard ratio	95% CI	*p*
Clinical T category	cT2	1.000 (reference)	—	—
	cT3	1.308	0.958–1.786	0.0914
	cT4a	1.894	1.380–2.599	< 0.0001
	cT4b	2.120	0.672–6.688	0.1993
Clinical N category	cN0	1.000 (reference)	—	—
	cN1	1.135	0.871–1.480	0.3488
	cN2	1.628	1.231–2.152	0.0006
	cN3	3.338	1.986–5.612	< 0.0001
Age, per 1‐year increase	—	1.056	1.042–1.071	< 0.0001
Sex	Female	1.000 (reference)	—	—
	Male	1.188	0.917–1.538	0.1925
Tumor location	Upper third	1.000 (reference)	—	—
	Middle third	0.919	0.689–1.226	0.5660
	Lower third	0.944	0.711–1.254	0.6910
Pretreatment histological type	Differentiated	1.000 (reference)	—	—
	Undifferentiated	1.390	1.096–1.763	0.0065

*Note:* Age was analyzed as a continuous variable, and the HR represents the increase in hazard per 1‐year increase in age. The multivariable Cox proportional hazards model included clinical T category, clinical N category, age, sex, tumor location, and pretreatment histological type.

Abbreviations: CI, confidence interval; HR, hazard ratio.

## 4 Discussion

This follow‐up study provides long‐term overall survival according to prospectively determined clinical T and N categories and their combinations in a multicenter cohort of patients with locally advanced gastric cancer. In this study, cT and cN stratified overall survival of patients with locally advanced gastric cancer, respectively; the 5 year‐OS was 82.1%/72.7%/60.0%/40.0% in cT2/cT3/cT4a/cT4b, and that was 78.0%/70.6%/59.1%/28.4% in cN0/cN1/cN2/cN3. In combination of cT and cN, 5y‐OS was 83.5% in cT2N0, 77.2% in cT3N0, 66.8% in cT4aN0, 78.7% in cT2N(+), 68.0% in cT3N(+), and 57.7% in cT4aN(+). The survival stratified by the combination of cT and cN has been previously reported by three studies [9, 10, 11]. The retrospective study with a large cohort from a single high‐volume center in Korea [9] showed the 5‐year overall survival as approximately 80% in cT2N0, 70% in cT3/T4aN0, 60% in cT3N+, and 50% in cT4aN+. In contrast to the present results, the survival curves for cT3N0 and cT4aN0 almost overlapped, whereas cT3N(+) was associated with clearly poorer survival than cT3N0 and cT4aN0. These differences may partly reflect the prospective, multicenter design of the present study, in which clinical staging was performed using prespecified diagnostic criteria. In the multivariable analysis adjusting for major pretreatment clinical factors, cT4a, cN2, and cN3 remained significantly associated with poorer overall survival, supporting the prognostic relevance of advanced clinical T and N categories after adjustment for these factors. These data provide a clinically relevant reference for estimating long‐term prognosis based on pretreatment clinical staging.

The results of this study showed that patients with locally advanced gastric cancer could be classified into three groups according to 5‐year overall survival. The most favorable group comprised cT2N0, cT2N(+), and cT3N0, with 5‐year overall survival of approximately 80%. The intermediate group comprised cT3N(+) and cT4aN0, with 5‐year overall survival of approximately 70%, whereas the poorest group comprised cT4aN(+) and cT4b disease, with 5‐year overall survival below 60%. The previous JCOG1302A study demonstrated substantial differences in the proportion of pathological Stage I disease among the clinical T/N subgroups. In the present follow‐up analysis, patients with pathological Stage I disease showed generally favorable long‐term survival across the preoperative clinical T/N subgroups, suggesting that the possibility of pathological Stage I disease should be taken into account when considering intensive perioperative treatment. Based on the survival outcomes observed in the overall cohort, cT3N(+), cT4aN0, cT4aN(+), and cT4b disease may represent clinically defined higher‐risk subgroups in whom perioperative treatment strategies warrant further consideration, although the findings for cT4b disease should be considered exploratory because of the very small number of patients in this subgroup. However, because the present study did not directly evaluate the efficacy of NAC, whether NAC provides a survival benefit in these specific clinical subgroups remains to be determined. Recently, the Pan‐Asian adapted ESMO Clinical Practice Guidelines [12] also emphasized that perioperative chemotherapy should be considered only for selected patients with locally advanced resectable gastric cancer, highlighting the importance of accurate clinical staging for optimal patient selection. Our multicenter prospective study provides long‐term prognostic information relevant to such patient selection. In contrast, in this study, both cT2N0 and cT3N0 patients demonstrated favorable long‐term survival even after upfront surgery‐based treatment, suggesting the importance of carefully balancing the potential benefit of perioperative treatment against its associated toxicity and treatment burden in these relatively favorable risk subgroups. In the context of recent advances such as the MATTERHORN trial [13], which showed improved outcomes with perioperative chemo‐immunotherapy, such approaches are associated with increased treatment‐related toxicity and higher economic cost. These findings suggest the importance of optimal patient selection when considering intensive perioperative treatment strategies.

In this study, the survival curves for cT3 and cT4a were clearly separated, which was concordant with previous studies [10, 11]. Bando et al. [10]. also demonstrated a substantial difference in survival between cT3 and cT4a disease. In the eighth edition of the TNM classification, however, cT3 and cT4a are grouped together in the clinical stage classification, despite the difficulty in distinguishing these categories clinically. Our results suggest that cT3 and cT4a disease distinguished by MDCT have different prognoses and may warrant separate consideration. This observation is also consistent with the exploratory radiological sub‐analysis of the PRODIGY trial [11], in which cT4 disease was associated with a minimal contamination of pathological Stage I disease and was identified as the subgroup achieving the greatest benefit from neoadjuvant chemotherapy, further supporting the clinical relevance of distinguishing cT4 from cT3 disease.

In this study, the survival curves for cN0 and cN1 were not clearly separated, which was inconsistent with the previous studies [9, 10]. The difference could be explained by the target cohort. cT1 was included in the previous studies but not in this study. The frequency of nodal metastasis was less than 20% in cT1 disease but exceeded 40% in cT2 or more advanced disease. In Japan and Korea, the frequency of cT1 disease was more than 50% among patients with gastric cancer. Thus, because previous studies included many cT1N0 patients, which would increase the survival of the cN0 category, the survival was separated between cN0 and cN1. In the combined cT/N analysis, cN1–3 disease was grouped as cN(+) to provide a clinically simple classification based on the presence or absence of clinically detected lymph node metastasis. However, the present study demonstrated progressively poorer survival from cN1 to cN3, indicating substantial prognostic heterogeneity within the cN(+) population. Therefore, the combined cT/N classification should be interpreted as a simplified approach for clinical risk stratification rather than as a replacement for the individual cN categories.

This study has several limitations for interpretation of the results. First, this study excluded patients who had Type 4 and large Type 3, so‐called scirrhous gastric cancer, and had paraaortic or extensive bulky lymph node metastasis. Thus, the present results could not be generalized to these patients. Second, the indication and regimen of adjuvant chemotherapy were not prespecified because of the observational nature of the study, and its administration varied among patients. Furthermore, the administration of adjuvant chemotherapy differed among clinical subgroups, which may have influenced the observed survival outcomes. Most patients with Stage III disease did not receive a doublet regimen such as S‐1 plus docetaxel because this study was conducted before the results of the JACCRO GC‐07 Phase III trial, which demonstrated the superiority of S‐1 plus docetaxel over S‐1 monotherapy [14], became available. This may also have negatively affected survival outcomes. Third, all patients were treated at specialized institutions in Japan, which should be considered when generalizing the present findings to broader patient populations. In addition, the survival data were obtained before perioperative chemo‐immunotherapy was established as a treatment option, and this difference in treatment era should be considered when interpreting the results. Finally, the numbers of patients with cT4b and cN3 disease were limited, resulting in wide confidence intervals; therefore, findings for these categories should be considered exploratory and interpreted with caution.

In conclusion, the present study clarified overall survival according to clinical T and N categories and their combinations, providing prognostic information that may help identify higher‐risk subgroups in whom perioperative treatment strategies warrant further investigation.

## Author Contributions

**Tsutomu Hayashi:** conceptualization, methodology, investigation, validation, writing – original draft, writing – review and editing, data curation. **Ryosuke Kato:** writing – review and editing, formal analysis. **Noriko Mitome:** formal analysis, writing – review and editing. **Rei Ogawa:** writing – review and editing, formal analysis. **Takeo Fukagawa:** conceptualization, methodology, writing – review and editing, data curation. **Hitoshi Katai:** conceptualization, methodology, writing – review and editing, data curation. **Souya Nunobe:** data curation. **Masanori Tokunaga:** data curation, writing – review and editing. **Etsuro Bando:** writing – review and editing, data curation. **Yuichi Ito:** writing – review and editing, data curation. **Takanobu Yamada:** writing – review and editing, data curation. **Takashi Nomura:** writing – review and editing, data curation. **Shigeto Makino:** writing – review and editing, data curation. **Takahiro Kinoshita:** writing – review and editing, data curation. **Hisashi Hara:** writing – review and editing, data curation. **Masaki Aizawa:** writing – review and editing, data curation. **Narikazu Boku:** writing – review and editing, conceptualization, project administration, data curation. **Yukinori Kurokawa:** project administration, writing – review and editing, data curation. **Masanori Terashima:** conceptualization, writing – review and editing, data curation. **Takaki Yoshikawa:** data curation, writing – review and editing, conceptualization, methodology.

## Funding

This work was supported by the National Cancer Center Research and Development Fund (2020‐J‐3, 2023‐j‐03, 2026‐j‐03).

## Ethics Statement

This study protocol was approved by the JCOG Protocol Review Committee and the institutional review board of each participating hospital before initiation of the study. This study was conducted in accordance with the international ethical recommendations stated in the Declaration of Helsinki and Ethical Guidelines for Medical and Health Research Involving Human Subjects.

## Conflicts of Interest

The authors declare no conflicts of interest.

## Supporting information


**Table S1:** Details of adjuvant chemotherapy according to combined clinical T and N categories.

## Data Availability

Data sharing not applicable to this article as no datasets were generated or analysed during the current study.
