# Supplementary material for: Survival Results by the Prospectively Determined Clinical Staging for Locally Advanced Gastric Cancer: A Follow‐Up Study of JCOG1302A
Source: Ann Gastroenterol Surg. 2026 Oct 6:10.1002/ags3.70293. Online ahead of print. doi: 10.1002/ags3.70293 (PMC13639646; doi:10.1002/ags3.70293)
Supplement: Supplementary file 1 [file AGS3-9999-0-s001.docx]

Supplementary Table. Details of adjuvant chemotherapy according to combined clinical T and N categories

| Clinical T/N subgroup* (Number of patients) | Number of patients (%) | | | | |
| --- | --- | --- | --- | --- | --- |
|  | Total** | S-1 | DS*** | Other | Unknown |
| cT2N0 (226) | 78 (34.5) | 69 | 2 | 7 | 0 |
| cT2N(+) (93) | 40 (43.0) | 34 | 0 | 6 | 0 |
| cT3N0 (232) | 105 (45.3) | 91 | 5 | 9 | 0 |
| cT3N(+) (218) | 135 (61.9) | 106 | 10 | 18 | 1 |
| cT4aN0 (100) | 58 (58.0) | 49 | 1 | 8 | 0 |
| cT4aN(+) (301) | 196 (65.1) | 172 | 3 | 21 | 0 |
| cT4bN0 (1) | 1 (100.0) | 1 | 0 | 0 | 0 |
| cT4bN(+) (5) | 2 (40.0) | 2 | 0 | 0 | 0 |
| Total (1176) | 615 (52.3) | 524 | 21 | 69 | 1 |

* Clinical T and N categories were based on the 14th edition of the Japanese Classification of Gastric Carcinoma

** Numbers in parentheses indicate the proportion of patients who received adjuvant chemotherapy

*** Docetaxel plus S-1
